# Conjoint Analysis of Genome-Wide lncRNA and mRNA Expression of Heteromorphic Leavesin Response to Environmental Heterogeneityin *Populus euphratica*

**DOI:** 10.3390/ijms20205148

**Published:** 2019-10-17

**Authors:** Ming Zeng, Shuhang He, Lin Hao, Yujie Li, Caixia Zheng, Yuanyuan Zhao

**Affiliations:** College of Biological Sciences and Technology, Beijing Forestry University, No. 35 Qing Hua Dong Lu, Beijing 100083, China; zengming1990@bjfu.edu.cn (M.Z.); shuhanghe@bjfu.edu.cn (S.H.); haolin@bjfu.edu.cn (L.H.); lyjbj1070@bjfu.edu.cn (Y.L.); zhengcx@bjfu.edu.cn (C.Z.)

**Keywords:** heterophylly, *Populus euphratica* Oliv., lncRNA-mRNA, light, environmental heterogeneity

## Abstract

Heterophylly is the phenomenon of leaf forms varying along the longitudinal axis within a single plant. *Populus euphratica*, a heterophyllous woody plant, develops lanceolate leaves and dentate broad-ovate leaves on the bottom and top of the canopy, respectively, which are faced with different intensities of ambient solar radiation. However, the mechanism of the heteromorphic leaf response to the microenvironment in *P. euphratica* remains elusive. Here, we show that the dentate broad-ovate leaves have advantages in tolerating high light intensity, while lanceolate leaves are excellent at capturing light. Compared with lanceolate leaves, more trichomes, higher stomatal density, thicker lamina, and higher specific leaf weight were observed in dentate broad-ovate leaves. Furthermore, high-throughput RNA sequencing analysis revealed that the expression patterns of genes and long noncoding RNAs (lncRNAs) are different between the two heteromorphic leaves. A total of 36,492 genes and 1725 lncRNAs were detected, among which 586 genes and 54 lncRNAs were differentially expressed. Based on targets prediction, lncRNAs and target genes involved in light adaption, protein repair, stress response, and growth and development pathways were differentially expressed in heteromorphic leaves, 10 pairs of which were confirmed by quantitative real-time PCR. Additionally, the analysis of interactions indicated that lncRNA–mRNA interactions were involved in the response to the microenvironment of heteromorphic leaves. Taken together, these results suggest that the morphological features and joint regulation of lncRNA–mRNA in heteromorphic leaves may serve as survival strategies for *P. euphratica*, which could lead to optimal utilization of environmental factors.

## 1. Introduction

Heterophylly, the phenomenon of leaf forms varying along the longitudinal axis of a single plant, results from the phenotypic plasticity of leaves and assists plants in adapting to environmental variations [1]. For this reason, heterophyllous plants are the perfect models for research on environmental adaptation [2,3]. Previous investigations have indicated that heterophylly contributes to acclimatizing plants to heterogeneous environmental factors, such as light, temperature, water, and so forth [2,3,4,5]. For example, in heterophyllous *Nuphar lutea* plants, owing to significant differences in the chloroplast ultrastructure, chlorophyll fluorescence, and pigment content, floating and aerial leaves were perceived to be sun adapted compared with submerged leaves [6]. In the study of *Rorippa aquatic*, heterophylly was identified as an adaption to the ambient temperature [7]. It was reported that *Hygrophila difformis* evolved heteromorphic leaves to deal with fluctuating water levels [8]. Furthermore, due to the striking environmental heterogeneity induced by water level, many aquatic and amphibious plants have developed heterophylly to acclimatize themselves to microclimate variations [1,8]. Additionally, some terrestrial plants, such as *Ginkgo biloba* and *Populus euphratica,* also exhibit heterophyllous characteristics [9,10,11], but as their habitat’s environmental heterogeneity seems less pronounced than that of aquatic and amphibious plants, their mechanism of heterophylly may be subtler. Therefore, a better understanding of the mechanism of heterophyllous woody plants using a molecular biological approach is necessary.

Long noncoding RNAs (lncRNAs) refer to transcripts that have a length of more than 200 (nucleotides (nt) and contain no apparent coding sequence (CDS) [12,13]. lncRNAs can modulate gene expression on various levels, by which biological pathways are finely tuned in plants to respond to stress and adapt to adverse conditions [14]. According to the genomic site relative to protein-coding genes, lncRNAs can be categorized into five groups: (a) intronic lncRNAs,(b) intergenic lncRNAs, (c)antisense lncRNAs, (d) sense lncRNAs, (e) bidirectional lncRNAs [15]. Generally, they regulate the transcriptional level of the target loci through cis-action or trans-action [16], showing obvious tissue-specific expression patterns and responses to environmental change [17]. lncRNAs have long been categorized as transcriptional “noise” because they cannot code for proteins. Recently, functions of lncRNAs have been identified in model plant species, such as Arabidopsis spp. [18], rice [19], maize [20], and *Populus* spp. [21]. In *Gossypium hirsutum*, 44 intergenic lncRNAs were differentially accumulated under salt stress, and controlled protein-coding genes via cis-acting regulation [22]. Moreover, downregulated polyadenylation (DPA) lncRNAs were found to be enriched in rice exposed to drought and salt, and they coexpressed with protein-coding genes related to stresses [23]. In tomato, lncRNA–mRNA networks have been established, and lncRNA16397 was identified to modulate *SlGRX* expression to reduce reactive oxygen species (ROS) accumulation, thus improving disease resistance [24]. Therefore, research on lncRNAs and coexpressed mRNAs could uncover the regulatory mechanism of biological processes in plants from a new angle, and progressive high-throughput RNA sequencing (RNA-Seq) technologies have provided a powerful approach for this objective [25].

*P. euphratica* Oliv., a representative heterophyllous woody plant, is mainly distributed in arid and semi-arid regions of China and has become a pioneer tree species in the area [26]. To efficiently adapt to environmental heterogeneity and achieve optimal utilization of resources, *P. euphratica* evolved heteromorphic leaves that vary from lanceolate leaves to dentate broad-ovate leaves distributed at the bottom and top of the crown in adult trees, respectively [27]. There have been many studies demonstrating that not only does the shape of the blades differ, but the structure and functional features of the blades also vary among heteromorphic leaves of *P. euphratica*. For example, previous studies demonstrated that dentate broad-ovate leaves are equipped with thicker cuticular wax than lanceolate leaves, which could protect the leaves from high solar radiation and excessive water loss by transpiration [26]. Recently, Hao et al. found that dentate broad-ovate leaves exhibit more distinct xeromorphic characteristics due to their thicker cell wall and higher mechanical strength compared with lanceolate leaves, which can support them to survive under arid and hot conditions [9]. Moreover, higher potential activity of photosystem II (PS II) and maximum photochemical efficiency of PS II were found in dentate broad-ovate leaves compared with lanceolate leaves, suggesting a higher photosynthetic capacity of dentate broad-ovate leaves to adapt to the higher intensity of solar light at the top of the canopy [28]. However, to date, studies of heteromorphic leaves in *P. euphratica* have mainly focused on structural and functional distinctions, and investigations of molecular regulation as well as the response to the microenvironment remain to be further elucidated. 

In the present study, morphological features of lanceolate leaves and dentate broad-ovate leaves, which are the two representative heteromorphic leaves of *P. euphratica,* were measured and compared. High-throughput lncRNA sequencing was employed to identify mRNAs and lncRNAs and to separately examine their expression patterns in these two types of leaves. Furthermore, based on the mechanism of cis-regulation or trans-regulationof lncRNAs, the interactions between differentially expressed genes (DEGs) and lncRNAs (DELs) were investigated to discern the role of lncRNAs in the regulation of gene expression, and the expression levels of these DEGs and DELs were validated by quantitative real-time PCR. In addition to this, the functions of lncRNAs of heteromorphic leaves in *P. euphratica* were explored according to the interactions. These results could reveal the regulatory roles of lncRNAs in response to the microenvironment of *P. euphratica* and provide new insights into the adaptation mechanism of environmental heterogeneity in heterophyllous plants.

## 2. Results

### 2.1. Morphological Feature of Two Heteromorphic Leaves

**Figure 1:**
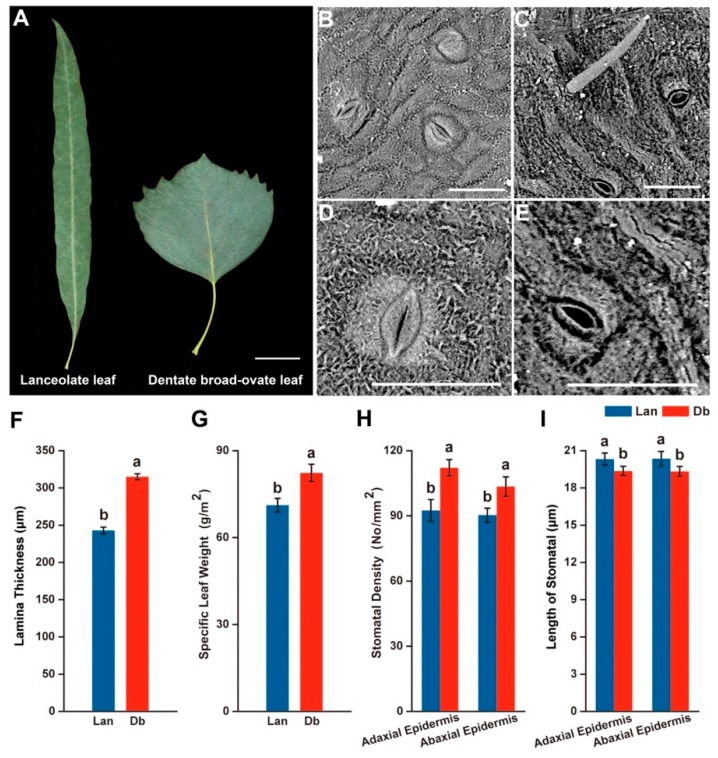
Morphological feature of the two typical heteromorphic leaves distributed at the bottom and top of the canopy of *Populus euphratica*. (**A**) The shape of lanceolate and dentate broad-ovate leaves (bar = 2 cm), (**B**) the epidermis of lanceolate leaf, (**C**) the epidermis of dentate broad-ovate leaf, (**D**) the stoma of lanceolate leaf, (**E**) the stoma of dentate broad-ovate leaf, (**F**) lamina thickness of lanceolate and dentate broad-ovate leaves, (**G**) specific leaf weight of lanceolate and dentate broad-ovate leaves, (**H**) stomatal density of lanceolate and dentate broad-ovate leaves, (**I**)length of stoma of lanceolate and dentate broad-ovate leaves. The data shown are the mean ± standard deviation; different letters indicate significant difference (*p* < 0.05) between samples (*t*-test listed in Table S1); scale bar of electron microscopy images = 50 μm; Lan, lanceolate leaves; Db, dentate broad-ovate leaves.

**Figure 2:**
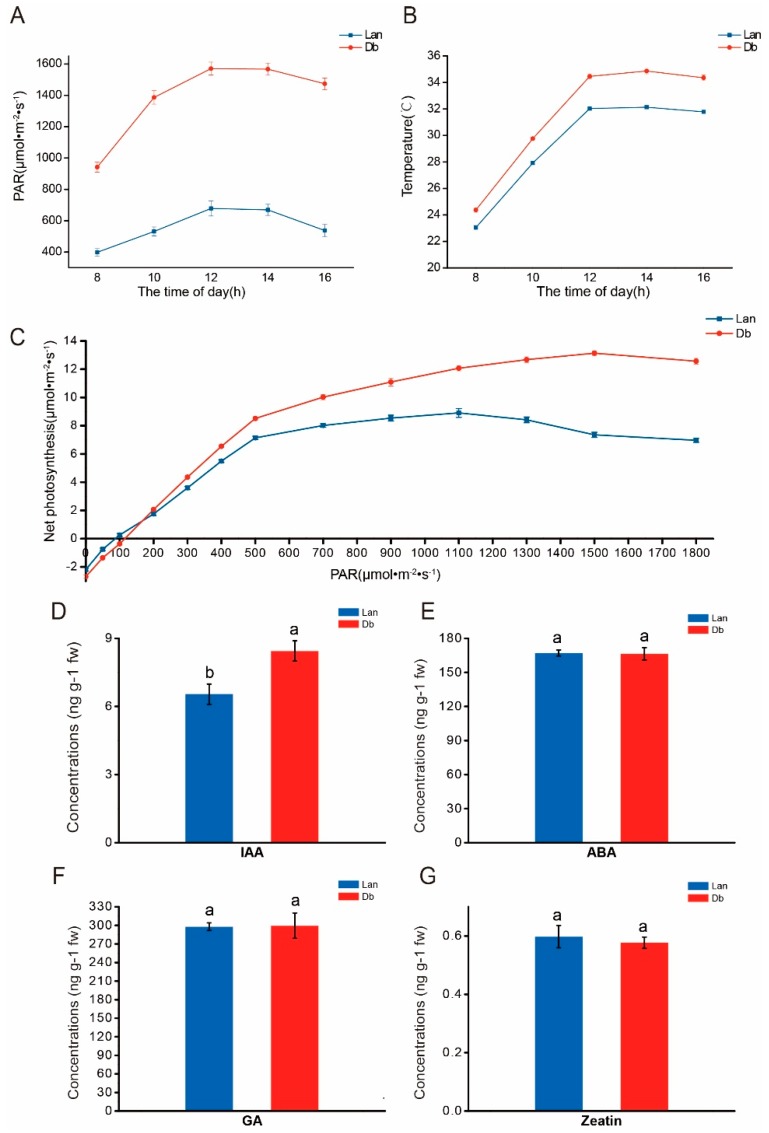
Environmental heterogeneity and phytohormone content of heteromorphic leaves in *P. euphratica*. (**A**) Intensity of light radiation on heteromorphic leaves, (**B**) leaf temperature of heteromorphic leaves, (**C**) net photosynthesis of heteromorphic leaves respond to different light intensity, (**D**) IAA content of heteromorphic leaves, (**E**) ABA content of heteromorphic leaves, (**F**) GA content of heteromorphic leaves, (**G**) zeatin content of heteromorphic leaves. Data are presented as mean ± SD; each experiment was performed with more than five replicates; different letters indicate significant difference (*p* < 0.05) between samples (*t*-test listed in Table S1); Lan, lanceolate leaves; Db, dentate broad-ovate leaves.

The shape of the lanceolate leaf was significantly different from that of the dentate broad-ovate leaf (Figure 1A). To examine the differences in morphological characteristics between the two types of leaves, scanning electron microscopy (SEM) was applied to inspect their epidermis. Trichomes developed on the epidermis of dentate broad-ovate leaves were observed (Figure 1C), while hardly any were observedon lanceolate leaves (Figure 1B). We further found that the stoma morphology between the two types of leaves was different (Figure 1D,E). The analysis results showed that the length of the stoma of dentate broad-ovate leaves was slightly shorter than that of lanceolate leaves (Figure 1I) (*t*-test listed in Table S1), while the stomatal density of dentate broad-ovate leaves was higher than that of lanceolate leaves both on the adaxial and abaxial epidermis (Figure 1H) (*t*-test listed in Table S1). In addition, we found that the laminas of dentate broad-ovate leaves were thicker than those of lanceolate leaves (Figure 1F) (*t*-test listed in Table S1), and the specific leaf weight(SLW) of dentate broad-ovate leaves was higher than that of lanceolate leaves (Figure 1G) (*t*-test listed in Table S1). These results suggest that there are obvious differences between lanceolate and dentate broad-ovate leaves in terms of epidermis, stoma, and lamina characteristics. In addition to measuring the morphological characteristics, we also investigated the microenvironment of the two types of leaves. As shown in (Figure 2A), the light intensity on dentate broad-ovate leaves was more than twice that of lanceolate leaves, which may result from their canopy distribution. The leaf temperature of dentate broad-ovate leaves was also higher than that of lanceolate leaves during the day, especially at noon; the temperature difference between the two leaves reached almost 3 °C at noon (Figure 2B). These results suggest that dentate broad-ovate leaves may undergo high radiation conditions coupled with high temperatures. 

To understand the response of heteromorphic leaves to light, the photosynthetic rates of the two types of leaves in different light intensities were determined. Generally, the light saturation point (LSP) represents the upper limit of the light intensity that the blade can use [29], while the light compensation point (LCP) reflects the leaf’s utilization of weak light [30]. Our data showed that the LSP of broad-ovate leaves was higher than that of lanceolate leaves, while the LCP of lanceolate leaves was lower than that of broad-ovate leaves (Figure 2C). Additionally, given that light can regulate the biosynthesis of phytohormones in plants [31], measurements were taken of the phytohormones in the two types of leaves. As presented in Figure 2D–F, the indole-3-acetic acid (IAA)content of dentate broad-ovate leaves was significantly higher than that of lanceolate leaves (*t*-test listed in Table S1), while the contents of abscisic acid (ABA), gibberellic acid (GA), and zeatin showed no obvious difference between the two types of leaves (*t*-test listed in Table S1). These results indicate that dentate broad-ovate leaves can efficiently perform photosynthesis under higher intensity light compared with lanceolate leaves, while the latter exhibits more efficient utilization of weak light. In addition, the difference in the light microenvironment between the two types of leaves may have an effect on their IAA contents.

### 2.2. Global Data Analysis of mRNA and lncRNA Expression in Heteromorphic Leaves

To obtain a comprehensive profile of the RNA expression, three biological repeats of lanceolate leaf (Lan) and dentate broad-ovate leaf (Db) samples were used for RNA-Seq, producing six strand-specific libraries (Lan_1, Lan_2, Lan_3, Db_1, Db_2, and Db_3). As shown in Table 1, for each replicate, over 160,000,000 raw sequence reads and 24 G raw bases were generated. After raw data trimming, more than 99,000,000 clean reads and 14.9 G clean bases were generated. The percentage of the GC content in dentate broad-ovate leaves was a slightly higher than that in lanceolate leaves. Then, the ribosomal RNA (rRNA) was removed, and nearly 75% of valid reads were mapped to the *P. euphratica* genome (https://www.ncbi.nlm.nih.gov/genome/?term=Populus%20euphratica) for each replicate (Table S2). The value of the Q20 proportion for all replicates wasmore than 99.6% (Table 1). In addition, the value of all the Pearson correlation coefficients between replicates exceeded 0.95 (Figure S1), indicating that the data from RNA sequencing were highly reliable.

**Figure 3:**
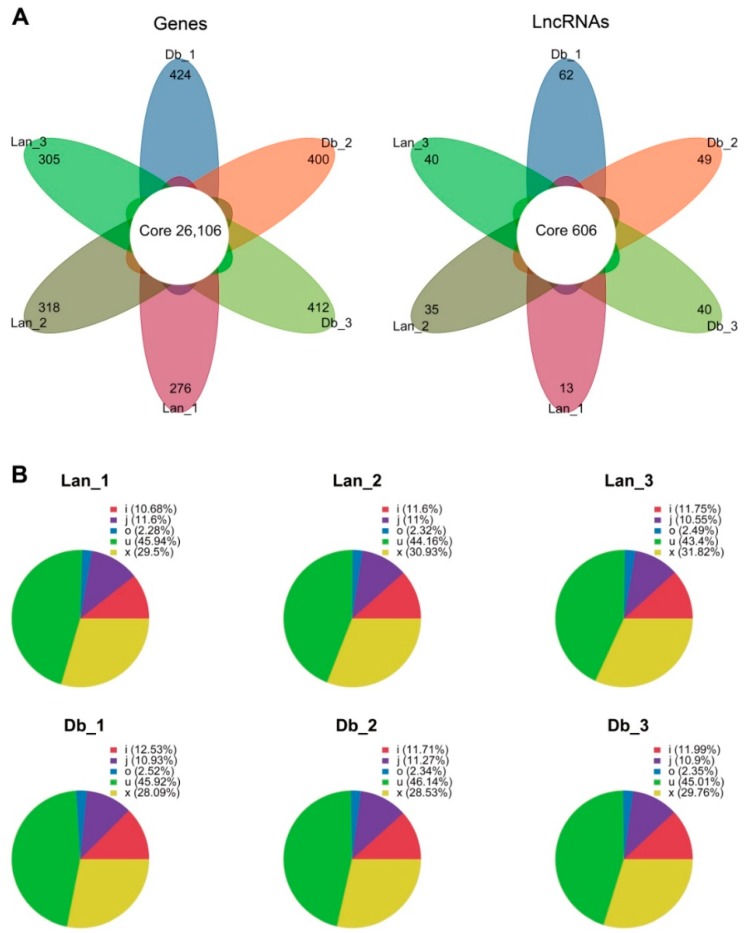
Status of genes and lncRNAs expressed in the six libraries. (**A**) Flower plot showing the numbers of genes and lncRNAs identified in the six libraries, (**B**) proportion of the different types of lncRNAs in the six libraries. Lan, lanceolate leaves; Db, dentate broad-ovate leaves. i (intronic lncRNA), j (bidirectional lncRNA), o (sense lncRNA), u (intergenic lncRNA), x (antisense lncRNA).

**Figure 4:**
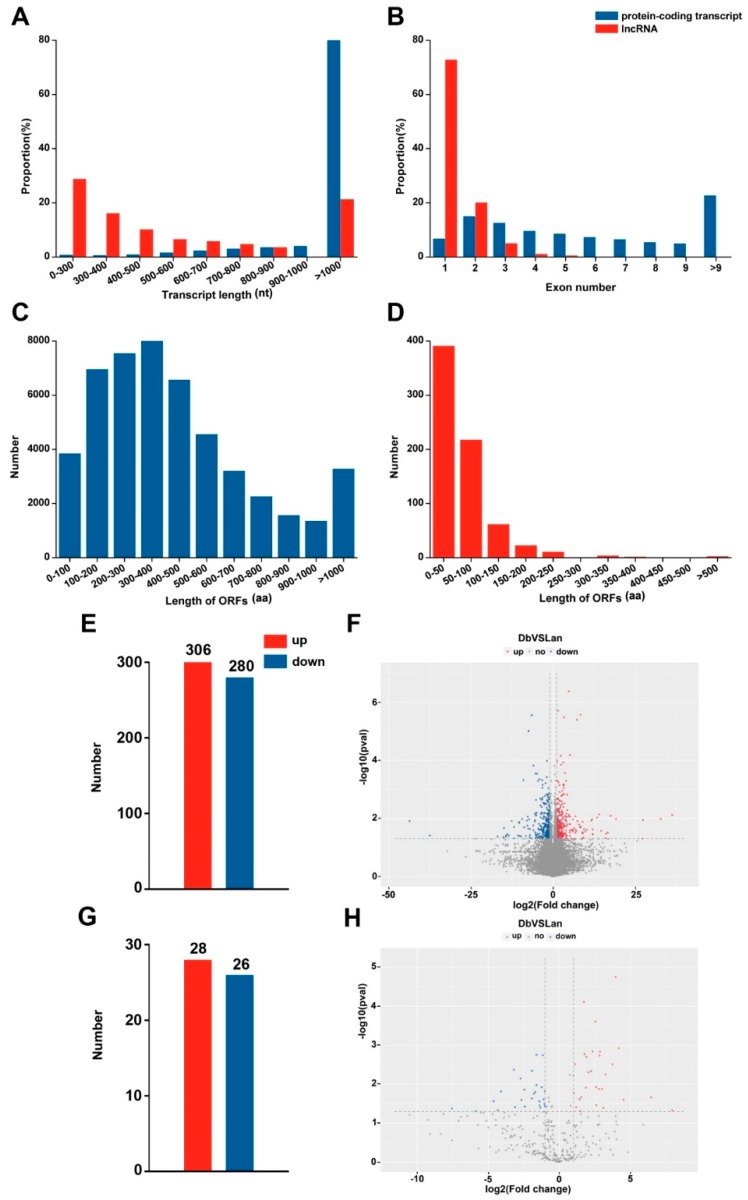
Comparisons of properties and differentially expressed mRNAs and lncRNAs between two heteromorphic leaves in *P. euphratica*. (**A**) Transcript length distribution of protein-coding transcripts and lncRNAs, (**B**) exon number of protein-coding transcripts and lncRNAs, (**C**) statistics of ORF length in protein-coding transcripts, (**D**) statistics of ORF length in lncRNAs, (**E**) the number of differentially expressed genes, (**F**) distribution of differentially expressed genes, (**G**) the number of differentially expressed lncRNAs, (**H**) distribution of differentially expressed lncRNAs. ORF, open reading frame; DEG, differentially expressed gene; DEL, differentially expressed lncRNA; Lan, lanceolate leaves; Db, dentate broad-ovate leaves.

After the sequences were assembled, a total of 36,492 genes (31,392 in Lan_1, 31,293in Lan_2, 31,410 in Lan_3, 31,399 in Db_1, 31,307 in Db_2, and 31,543 in Db_3) were obtained from the six libraries (Table 2). Correspondingly, a total of1725 lncRNAs (1095 in Lan_1, 1164 in Lan_2, 1166 in Lan_3, 1189 in Db_1, 1153 Db_2, and 1193 in Db_3) were generated through the screening process. Except for a few genes that were specifically expressed in different samples, 26,106 genes were expressed in all replicates of heteromorphic leaves, and 606 lncRNAs were identified in all of these replicates (Figure 3A). The regulatory effect of a lncRNA is usually associated with its relative position to a protein-coding gene [15]. Accordingly, the lncRNAs identified in the two types of leaves were divided into five categories: intergenic lncRNA (coded as u), intronic lncRNA (coded as i), bidirectional lncRNA (coded as j), sense lncRNA (coded as o), and antisense lncRNA (coded as x). Figure 3B showed that intergenic lncRNA was the largest component, accounting for almost half of all lncRNAs in the two heteromorphic leaves. The investigation of the sequence length demonstrated that 82% of protein-coding transcripts were longer than 1000 bp, while only a small portion of lncRNAs (21%) was longer than 1000 bp (Figure 4A). This suggests that lncRNAs are shorter than protein-coding transcripts in *P. euphratica*. Moreover, compared with the protein-coding transcripts, lncRNAs contained fewer exons, 72% of which contained only one exon, while most of the protein-coding transcripts (79%) had more than two exons (Figure 4B). We further found that the length of open reading frames (ORFs) in 85% of lncRNAs were shorter than 100 aa, while those in only 8% of protein-coding transcripts were shorter than 100 aa, indicating longer ORFs in protein-coding transcripts compared with lncRNAs (Figure 4C,D). These results showed the differential expression pattern between protein-coding transcripts and lncRNAs in *P. euphratica.*

### 2.3. Identification of Differentially Expressed Genes and lncRNAs 

**Figure 5:**
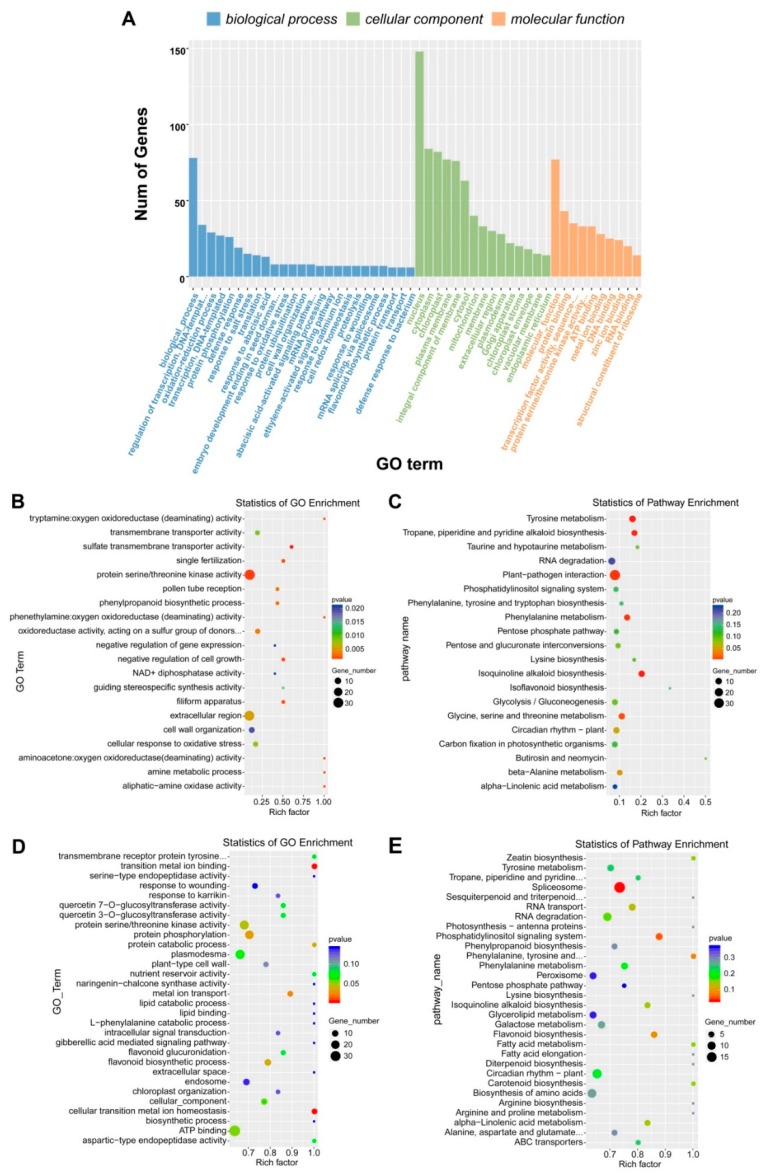
GO and KEGG pathways analysis of differentially expressed genes and target genes between two heteromorphic leaves in *P. euphratica*. (**A**) GO annotation of differentially expressed genes between lanceolate and dentate broad-ovate leaves, (**B**) statistics of GO enrichment of differentially expressed genes, (**C**) statistics of KEGG pathway enrichment of differentially expressed genes. (**D**) statistics of GO enrichment of differentially expressed target genes of differentially expressed lncRNAs, (**E**) statistics of KEGG pathway enrichment of differentially expressed target genes of differentially expressed lncRNAs.

To investigate the differences in the expression level of genes and lncRNAs between lanceolate and dentate broad-ovate leaves, the fragments per kilobase per million mapped reads (FPKM) method was used to measure the expression level. Genes or lncRNAs with |log2 (fold change)| ≥ 1 and with statistical significance (*p*-value < 0.05) were considered as differentially expressed genes (DEGs) or differentially expressed lncRNAs (DELs). Comparing dentate broad-ovate leaves with lanceolate leaves, a total of 586 DEGs (Additional file 1) were discovered, including 306 upregulated DEGs and 280 downregulated DEGs (Figure 4E,F). Moreover, 28 upregulated DELs and 26 downregulated DELs were found in the comparison (Figure 4G,H), so the total number of DELs was less than that of DEGs. Gene Ontology (GO) annotation was further applied to evaluate the functions of DEGs which were classified into “biological process”, “cellular component” and “molecular function” categories (Figure 5A). There are functional differences between lanceolate leaves and dentate broad-ovate leaves in responding to the microenvironment. To decipher biological processes that involved in response to the microenvironment, GO enrichment of DEGs was performed. A number of DEGs were identified to be associated with “protein serine/threonine kinase activity” and “extracellular region” which could accept signals from receptors that perceive microenvironment and transform it into proper outputs such as regulation in metabolism. As the results of the enrichment analysis of GO annotation of DEGs shown in Figure 5B, DEGs were mainly significantly enriched in “protein serine/threonine kinase activity” (33 DEGs) and “extracellular region” (30 DEGs) GO terms. Furthermore, by performing the enrichment analysis of Kyoto Encyclopedia of Genes and Genomes (KEGG) pathways, a number of DEGs related to amino acid metabolism, secondary metabolism, plant-pathogen interaction were identified in the two types of leaves, which could be involved in metabolism regulation and signal transduction of response to microenvironment in heteromorphic leaves. As shown in Figure 5C, DEGs were primarily significantly enriched in “plant–pathogen interaction”(36 DEGs);“tyrosine metabolism”(11 DEGs); “isoquinoline alkaloid biosynthesis”(10 DEGs); and “glycine, serine, and threonine metabolism”(10 DEGs) pathways. These results indicate that genes are differentially expressed between the two types of heteromorphic leaves, which could be mainly involved in protein serine/threonine kinase activity, amino acid metabolism, secondary metabolism, and plant–pathogen interaction pathways in responding to the microenvironment in *P.euphratica*.

### 2.4. Identification of Differentially Expressed lncRNA-mRNA Interaction Pairs

To determine the function of differentially expressed lncRNAs and their potential target mRNAs, interaction pairs of lncRNAs and mRNAs were predicted based on cis-acting and trans-acting regulation patterns. The results showed that 487 differentially expressed mRNA were identified as potential targets for the 50 differentially expressed lncRNAs in heteromorphic leaves of *P. euphratica* (Additional file 2). Then, based on the enrichment analysis of GO annotation, we identified a number of target genes associated with ion homeostasis and protein phosphorylation, which are essential for plant growth regulation and environmental response. As shown in Figure 5D, the differentially expressed target genes were mainly enriched in “transition metal ion binding”, “cellular transition metal ion homeostasis”, and “protein phosphorylation” GO terms. Furthermore, by performing the enrichment analysis of the KEGG pathway, a number of target genes related to signal transduction, photosynthesis, and plant circadian rhythm were identified in heteromorphic leaves, which may play important roles in responding to the microenvironment, especially the light environment. As shown in Figure 5E, the target genes were mainly enriched in the “spliceosome”, “phosphatidylinositol signaling system”, “circadian rhythm−plant”, and “photosynthesis−antenna proteins” pathways. These results demonstrate that the differentially expressed lncRNA–mRNA interaction pairs may participate in photosynthesis and plant circadian rhythm pathways which could be affected by the light condition, to respond to the microenvironment.

### 2.5. Interaction of lncRNAs and Target Genes Response to Microenvironment of Heteromorphic Leaves

**Figure 6:**
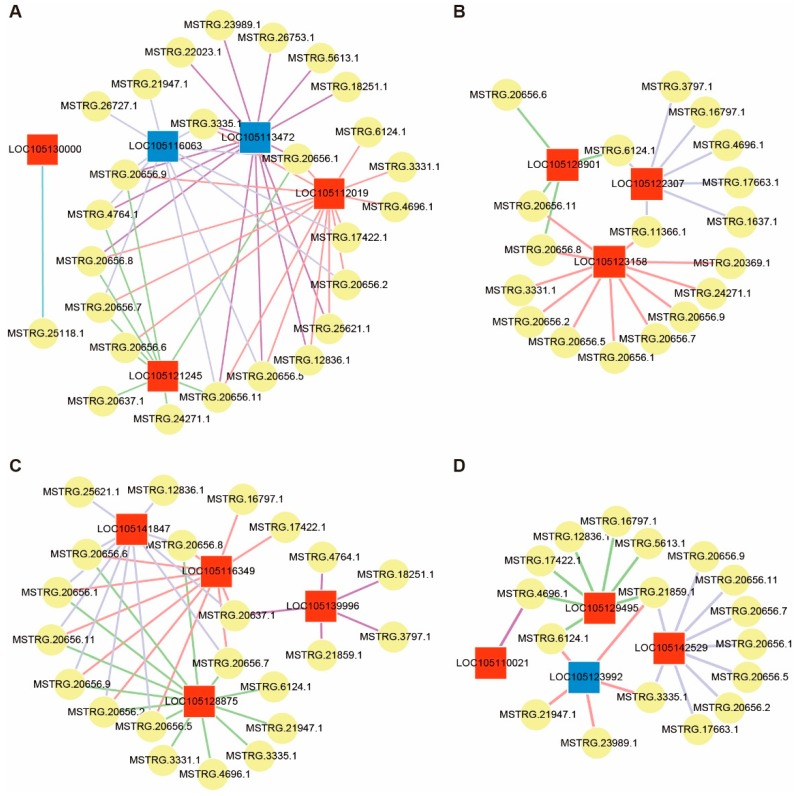
The lncRNAs and target protein-coding gene interactions related to adaption. (**A**) Interactions associated with light adaption, (**B**) interactions associated with protein repair, (**C**) interactions associated with stress response, (**D**) interactions associated with growth and development. The circular and square nodes represent differentially expressed lncRNAs and differentially expressed target genes, respectively. The red and blue square nodes represent upregulated and downregulated genes, respectively. Details of the interaction relationships among lncRNAs and target protein-coding genes are shown in Additional file 3.

lncRNAs could be involved in the response to different microenvironments through regulating their potential target genes. Based on the functional enrichment of target genes described above, we focused on the differentially expressed target genes related to light, stress response, protein repair, and growth and development functional clusters (Additional file 3). Furthermore, in order to visualize the interaction relationship, networks were constructed by using Cytoscape software (Figure 6). Among the interactions, most of the potential target genes were regulated by several lncRNAs, while very few genes were merely potentially regulated by one lncRNA.

Given that there were significant differences in light intensity between the two heteromorphic leaves (Figure 2), we therefore analyzed the lncRNA–mRNA interaction pairs involved in the response to light. In this functional cluster, five differentially expressed genes were predicted as the target genes of 26 differentially expressed lncRNAs, and their expression changes showed both positive and negative correlation relationships in the interactions (Additional file 3). Among these five target genes, two genes (LOC105130000 and LOC105121245) involved in the wax biosynthesis process were obviously upregulated, and one gene (LOC105112019) involved in xanthophyll cycle-dependent photoprotection was upregulated; the remaining two genes (LOC105113472 and LOC105116063) associated with antenna proteins of photosynthesis were down-regulated. Furthermore, we found that LOC105130000 (3-ketoacyl-CoA synthase 11-like) was regulated by only one DEL (MSTRG.25118.1), while other genes interacted with more than nine DELs (Figure 6A).

To acclimatize to various living environments, plants, as sessile organisms, have evolved accurate and complex stress response mechanisms over their long-term evolution. As to the cluster of stress response, four genes related to stress response were significantly upregulated, including LOC105128875 (peptidyl-prolyl cis-trans isomerase), LOC105116349 (WRKY transcription factor 6-like), and genes (LOC105139996 and LOC105141847) of two leucine-rich repeat receptor-like protein kinases, which were identified as the putative target genes of 22 differentially expressed lncRNAs (Additional file 3). Each of these up-regulated genes was targeted by at least five DELs (Figure 6C). In addition, the protein repair system is important for plants to sustain their normal functions under disadvantageous conditions, such as the high-light and high-temperature environments which dentate broad-ovate leaves may encounter (Figure 2A,B). For the interactions of protein repair, 3 up-regulated genes (LOC105123158, LOC105122307, and LOC105128901) closely interacted with 18 differentially expressed lncRNAs (Additional file 3), 11 of which were up-regulated and 9 were down-regulated in the comparison of dentate broad-ovate to lanceolate leaves (Figure 6B). Moreover, all three upregulated genes were involved in the cysteine and methionine metabolism pathway, which plays a vital role in repairing reversible modifications of proteins in plants.

Genes related to phytohormones and circadian rhythm, which can regulate the growth and development of plants, may play important roles in the regulation of heteromorphic leaves. In terms of the growth and development cluster, four differentially expressed genes associated with the growth and development process closely interacted with 17 differentially expressed lncRNAs (Figure 6D). Among these protein-coding genes, one *AUX/IAA* gene (LOC105129495) and two *CONSTANS-LIKE* genes (LOC105142529 and LOC105110021) were up-regulated, while one *CONSTANS-LIKE* gene (LOC105123992) was down-regulated (Additional file 3).

### 2.6. Validation of qRT-PCR

**Figure 7:**
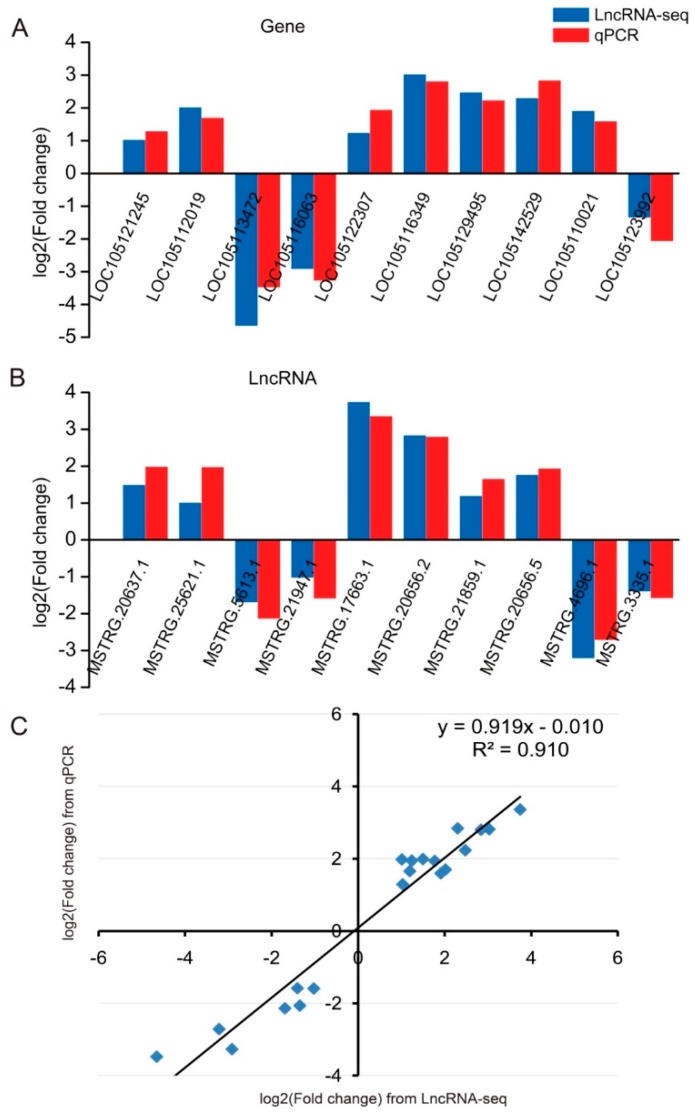
qRT-PCR validation of the candidates from lncRNAs and target protein-coding gene interactions. (**A**) qRT-PCR validation of 10 selected genes from lncRNAs and target protein-coding gene interactions, (**B**) qRT-PCR validation of 10 selected lncRNAs from lncRNAs and target protein-coding gene interactions, (**C**) coefficient analysis for expression fold change of the validation candidates from lncRNA-seq and qRT-PCR.

Based on functional analysis of target genes and lncRNAs, 10 DEGs and 10 DELs were randomly selected for qRT-PCR validation using the same samples as for RNA-seq to confirm the data of gene and lncRNA expression. The primers of DEGs and DELs are exhibited in Table S3. The expression levels of seven target genes (LOC105121245, LOC105112019, LOC105122307, LOC105116349, LOC105129495, LOC105142529, and LOC105110021) that relate to the biosynthesis of wax, photoprotection, growth and development process, and stress response were higher in dentate broad-ovate leaves than in lanceolate leaves. The expression of two genes (LOC105113472 and LOC105116063) of chlorophyll a/b-binding protein, which is associated with light energy capture in photosynthesis, and a *COL 6* gene were markedly lower in dentate broad-ovate leaves compared with lanceolate leaves (Figure 7A). In addition, the expression levels of six candidate lncRNAs were significantly higher in dentate broad-ovate leaves than in lanceolate leaves, whereas the remaining four candidate lncRNAs exhibited lower expression levels in dentate broad-ovate leaves compared with lanceolate leaves (Figure 7B). Overall, the results showed that the expression profiles of the candidate genes and lncRNAs obtained from qRT-PCR analysis were relatively consistent with those from the high-throughput RNA sequencing (Figure 7A,B). Additionally, a significantly positive correlation was found in the linear regression analysis between the expression changes (DEGs and DELs) determined by lncRNA-seq and qRT-PCR (Figure 7C). These results indicate that the profiles of gene and lncRNA expression from high-throughput RNA sequencing are reliable, and they further confirm the differences in biosynthesis of wax, photoprotection, light-harvesting, growth and development process, and stress response between the two heteromorphic leaves.

## 3. Discussion

### 3.1. Heteromorphic Leaves Exhibit Different Morphological and Physiological Features to Respond to the Heterogeneous Microenvironments

Leaf variations induced by phenotypic plasticity play a crucial part in environmental adaptation and resource utilization for plants [32]. When leaf variations occur within a single plant, this is known as heterophylly [1]. Although single-plant heteromorphic leaves form within the same environmental habitat, their respective microenvironments may vary along the longitudinal axis of the plant [3]. Previous studies found that in some aquatic and amphibious plants, heteromorphic leaves evolved as a survival strategy, where the different relative positions to the water surface caused microenvironmental heterogeneity [2,8]. These heteromorphic leaves exhibit differences not only in leaf shape but also in morphological and physiology features [7]. In terrestrial plants, microenvironmental heterogeneity also exists, but it is not as obvious as that of aquatic and amphibious plants [1]. Even so, heterophylly has also evolved in some terrestrial plants, especially woody plants such as *P. euphratica* [9], *Syringa oblate* [11], and *G. biloba* [10]. In the present study, we found that the shapes of lanceolate and dentate broad-ovate leaves in *P. euphratica* were quite different (Figure 1A). We further showed that, compared with lanceolate leaves, dentate broad-ovate leaves were exposed to higher solar radiation due to their upper canopy position (Figure 2A).Further, the leaf temperature of dentate broad-ovate leaves was also higher than lanceolate leaves in the daytime (Figure 2B), which may be influenced by the different intensities of solar radiation on the blades, indicating that environmental heterogeneity exists in heteromorphic leaves of *P. euphratica*.

Microenvironmental changes may bring about variations of leaves, both in terms of morphology and physiology [7,33]. TATTINI et al. demonstrated that trichomes are efficient at attenuating excess solar irradiance [34]. In the present study, trichomes were mainly observed on the epidermis of dentate broad-ovate leaves (Figure 1B,C), which could protect this type of leaf from the high solar radiation at midday. Previously, decreased stomatal length and increased stomatal density were observed in alfalfa under adverse environmental conditions [35]. Here, we found that, compared with lanceolate leaves, the stomata size was smaller and the stomata density was higher in dentate broad-ovate leaves (Figure 1H,I), which may be conducive to efficiently regulating water loss by transpiration, especially under the relatively higher light intensity and temperature conditions. It has also been reported that increased SLW and leaf thickness are regarded as a response to high light radiation [11,36], which was also observed in the dentate broad-ovate leaves in our study. Conversely, a lower SLW usually exists in shade plants as a shade tolerance strategy [37]. For example, the SLW of shade trees was found to be significantly lower than that of sun trees in *Syringa oblata* [11]. Similarly, we found that the SLW was lower in lanceolate leaves compared with dentate broad-ovate leaves, indicating that the investment per unit of light capture surface area of these bottom leaves was less than that of the top leaves in *P. euphratica*. In addition, by comparing five contrasting trees species, Urban et al. found that sun trees had significantly higher maximum net photosynthetic rates than shade trees [38]. Through the investigation of trends in net photosynthetic rate of the two heteromorphic leaves in different light intensities (Figure 2C), we found that higher intensity light could be utilized in dentate broad-ovate leaves compared with lanceolate leaves. These results suggest that heteromorphic leaves exhibit differences in morphological and physiological properties as a response to the heterogeneous microenvironment of *P. euphratica*, which may be a delicate survival mechanism for this heterophyllous woody plant.

### 3.2. Differential Expression Pattern of Genes and lncRNAs in Heteromorphic Leaves

It has been reported that gene expression regulation plays a crucial role in environmental adaptation in plants [39,40]. Recently, the expression pattern of lncRNA and its regulatory functions have received more attention, and some research has demonstrated that lncRNAs can regulate the expression of target genes involved in the response of plants to their surroundings [22,41,42]. Our high-throughput RNA sequencing results revealed that nearly 120,000,000 and 110,000,000 valid data were generated from lanceolate leaves and dentate broad-ovate leaves in *P. euphratica*, respectively (Table 1). The abundant valid data provided by the high-throughput sequencing allowed us to comprehensively survey gene and lncRNA expression in heteromorphic leaves, and 36,492 genes and 1725 lncRNAs were detected in *P. euphratica* (Table 2). The number of lncRNAs obtained in this research was more than that which was determined in *Populus tomentosa* [21], which may be due to species-specific of lncRNA. We found that lncRNAs possess a shorter transcript length and fewer exons compared with protein-coding transcripts in *P. euphratica* (Figure 4A,B), which are findings that are similar to previous results for other species, such as *Cleistogenes songorica* [42], *Ginkgo biloba* [43], and *Medicago truncatula* [44]. Previously, He et al.identified6822 differentially expressed genes between different samples of *Potamogeton octandrus*, a heterophyllous aquatic plant [4]. In our investigation, 586 differentially expressed genes and 54 differentially expressed lncRNAs were identified between the two heteromorphic leaves (Figure 4E,G), implying their specific expression pattern in *P. euphratica*, which may contribute to the response to environmental heterogeneity of these two heteromorphic leaves distributed in different layers of the canopy in *P. euphratica.*

### 3.3. lncRNA–mRNA Interaction Involved in Response to Light

Plant leaves absorb light for photosynthesis, but there are differences in terms of the light intensity on leaves in different layers of the canopy [45]. Similarly, this situation was also found in the present study of *P. euphratica* (Figure 1), where the light intensity on the top canopy was much higher than that on the bottom canopy. Notably, excess light may damage leaves. To maintain normal physical activity, plants have evolved some protection systems to respond to high light intensity [46,47]. For example, cuticular waxes serve as the first barrier protecting leaves from excess radiation, which could limit light transmittance [48], and mainly consist of very long-chain hydrocarbon compounds [49,50]. Biosynthesis of cuticular waxes starts from fatty acid (FA) elongation, which is regulated by the rate-limiting enzyme 3-KETOACYL-CoA SYNTHASE (KCS) [51]. Previous research indicated that leaf wax crystal formation is positively correlated with the expression level of the *KCS* gene in rice [52]. In this study, we found that the expression of *KCS* (LOC105130000), targeted by lncRNAs (MSTRG.21896.1 and MSTRG.25118.1) (Figure 6A), was obviously upregulated, which may increase the content of cuticular waxes in dentate broad-ovate leaves. Moreover, the encoding gene (LOC105121245) of protein ECERIFERUM 3 (CER3), which has been proven to promote the biosynthesis of leaf wax after the FA elongation process [53], was also upregulated in dentate broad-ovate leaves and was targeted by differentially expressed lncRNAs (MSTRG.20637.1, MSTRG.4764.1, MSTRG.20656.1, etc.) (Figure 6A). Consistent with previous investigations of the molecular mechanism and function of cuticular wax [54,55], the expression levels of these genes (LOC105130000 and LOC105121245) related to wax biosynthesis were obviously up-regulated in dentate broad-ovate leaves. Therefore, we propose that the increase of wax biosynthesis could be one form of plant photoprotection mechanism in *P. euphratica*.

**Figure 8:**
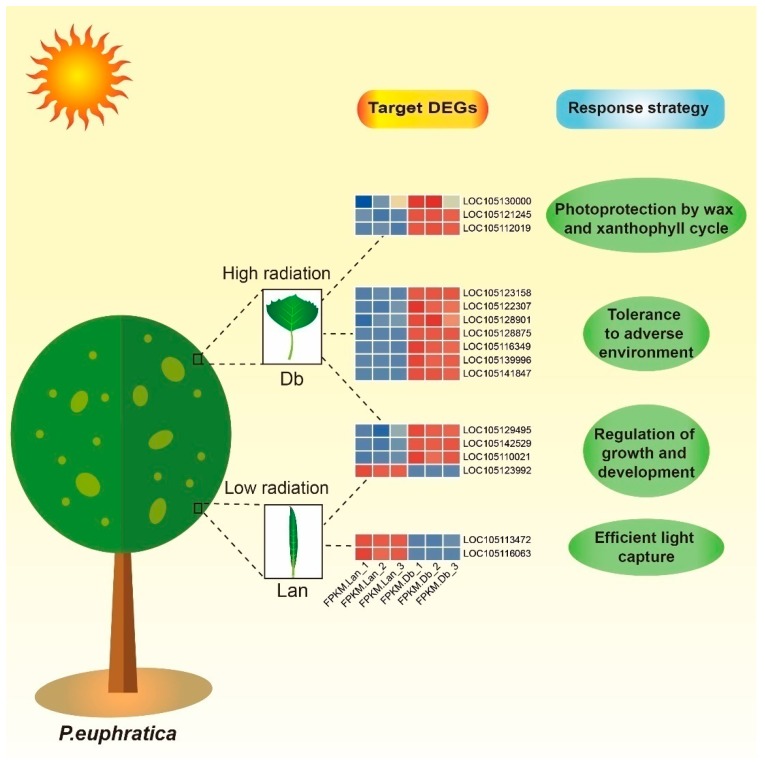
The response to the environmental heterogeneity of heteromorphic leaves in *P. euphratica*. Data for the gene expression level were normalized to Z-scores. DEGs, differentially expressed genes; Lan, lanceolate leaves; Db, dentate broad-ovate leaves.

For higher plants, xanthophyll cycle-dependent photoprotection is an efficient pathway to dissipate surplus light energy to avoid photodamage [56], and zeaxanthin epoxidase serves as the rate-limiting enzyme in the xanthophyll cycle [57]. Here, the expression of the encoding gene (LOC105112019) of zeaxanthin epoxidase was significantly up-regulated in dentate broad-ovate leaves (Additional file 3), suggesting that this type of leaf may have advantages in the xanthophyll cycle to dissipate excess radiation (Figure 8). In addition, the available light resources for lanceolate leaves are limited due to their distribution on the bottom of the canopy. Thus, they need to capture as much light as possible to perform photosynthesis. Regarding *Arabidopsis*, Umate et al. reported that the antenna protein is known to play a vital role in light harvesting for photosynthesis [58]. In the present study, two encoding genes (LOC105113472 and LOC105116063) of the chlorophyll a/b-binding protein, which is a type of antenna protein in plants [58], were both found to be upregulated in lanceolate leaves. This result indicates that lanceolate leaves may be more efficient at light harvesting, which helps them make full use of the lower solar radiation (Figure 8). Together, these results suggest that the xanthophyll cycle and light-harvesting systems are enhanced in dentate broad-ovate leaves and lanceolate leaves, respectively, to respond to their light microenvironment.

### 3.4. lncRNA–mRNA Interaction Involved in Response to Adverse Environment

Commonly, high-light and high-temperature conditions lead to excess ROS accumulation in leaves and further cause oxidative stress [59]. Oxidative stress may lead to deleterious modifications of proteins [60], which can be classified into reversible and irreversible forms [61,62], but plants have evolved protective systems that allow them to repair these modifications. Reversible modifications merely occur in methionine and cysteine, since these two sulfur-containing amino acids can be reduced by a cellular antioxidative process [63]. In the present study, the expression levels of three crucial protein-coding genes (LOC105123158, LOC105122307, and LOC105128901) in cysteine and methionine metabolism pathways were all significantly upregulated in dentate broad-ovate leaves, which endure high-light and high-temperature conditions, and potentially targeted by interaction DELs (MSTRG.3331.1, MSTRG.6124.1, MSTRG.20656.6, etc.) (Figure 6B). Taken together, we conclude that cysteine and methionine metabolism may play an important role in heteromorphic leaves of *P. euphratica* to deal with reversible protein modification.

Under adverse environmental conditions, peptidyl-prolyl cis-trans isomerase, a heat-induced enzyme, can assist plants to maintain the protein’s natural conformation [64,65]. In the present study, our results showed that the expression of the encoding gene (LOC105128875) of peptidyl-prolyl cis-trans isomerase was significantly upregulated in dentate broad-ovate leaves, which withstand higher temperatures in the upper canopy, indicating that this enzyme may be crucial for dentate broad-ovate leaves to maintain the protein’s natural conformation. Previously, the plant-specific transcription factor family WRKY was identified to be the vital transcription factor that regulates abiotic stress tolerance in plants at the post-transcriptional level [66]. It was observed that overexpression of *WRKY 39*, a heat-induced *WRKY* member, enhances thermotolerance in transgenic *Arabidopsis thaliana* [67]. In our study, the expression level of the gene WRKY transcription factor 6 (LOC105116349) was obviously increased indentate broad-ovate leaves, indicating that *WRKY 6* may be induced by high-radiation and/or high-temperature environments. Furthermore, it was reported that lncRNA–mRNA interactions were involved in responses to some adverse conditions in plants [22,68]. By further analysis of lncRNA–mRNA interaction, we found that the expression levels of two encoding genes (LOC105139996 andLOC105141847) of leucine-rich repeat receptor-like protein kinases (LRR-RLKs), which are involved in plant stress responses [69,70], were both increased in dentate broad-ovate leaves. The above results indicate that the interaction of these DELs and their targets could play a crucial role in the response to adverse environments in dentate broad-ovate leaves of *P. euphratica*. Thus, we propose that a higher tolerant ability is exhibited in dentate broad-ovate leaves under adverse environmental conditions compared with lanceolate leaves (Figure 8), which agrees with previous investigations of heteromorphic leaves of *P. euphratica* [9,26,71].

### 3.5. lncRNA–mRNA Interaction Involved in Growth and Development

Light is not only an energy resource for plants but also a crucial environmental signal influencing plant growth and development [72]. Light signals can affect the circadian rhythm of plants to regulate their growth and development [73]. In this process, CONSTANS-LIKE (COL) is a vital modulator for the connection between the light signal and circadian clock to form a control for plant development. In this study, the expression of *COL 2* (LOC105142529) and *COL 11* (LOC105110021) were significantly increased in dentate broad-ovate leaves, while the expression level of COL 6 (LOC105123992) was downregulated. The further phylogenetic analysis showed that there was a close phylogenetic relationship between *COL 2* and *COL 11*, whereas *COL 6* was distantly phylogenetically related to them (Figure S2), implying that there are differences in their biological functions. Previous studies have demonstrated that COL transcription factors can regulate the growth and development process of flowers [74] and roots [75]. In contrast, we found that these *COL* genes were differentially expressed in heteromorphic leaves, suggesting that the regulators of circadian rhythm may also influence the growth and development of heteromorphic leaves induced by light heterogeneity in *P. euphratica* (Figure 8). Furthermore, it was reported that light could affect the biosynthesis of phytohormones [31]. For example, the Auxin IAA, the biosynthesis of which is affected by light conditions, could participate in the growth and development processes of plants [76]. In our investigation, the examination of phytohormones showed that the auxin content was significantly higher in dentate broad-ovate leaves than in lanceolate leaves (Figure 2D). Moreover, the analysis of the RNA-sequence showed that the expression of the *AUX/IAA* gene (LOC105129495) was significantly increased in dentate broad-ovate leaves, which was targeted by DELs (MSTRG.6124.1, MSTRG.5613.1, etc.) (Figure 6D). Overall, these results imply that the circadian rhythm and the auxin signaling pathway, which are induced by light signals, may be involved in the growth and development of heteromorphic leaves in *P.euphratica*.

## 4. Materials and Methods

### 4.1. Plant Materials

The samples used in this study were collected from adult *P. euphratica* Oliv. trees growing under natural conditions at Beijing Forestry University, northwestern Beijing, China. *P. euphratica* simultaneously generate numerous types of leaf shapes within an individual plant, and the two typical heteromorphic leaves located at the bottom and the top of the crown are lanceolate leaves and dentate broad-ovate leaves (Figure 1A), referred to as Lan and Db samples, respectively, in our study. All of the leaves obtained from every tree were healthy, mature, and unbroken.

### 4.2. Measurement of Leaf Morphological and Physiological Features

To survey the SLW of the heteromorphic leaves (Figure 1A), 15 blade discs with a given area per piece were obtained from mature and integrated leaves by a borer. Then, these blade discs were dried at 60 °C for 30 h, after which constant weight determination was conducted with an electron balance. The SLW was calculated by dividing the leaf dry weight by the relevant leaf area (g/m^2^) [77]. The two heteromorphic leaf tissues were collected from three *P. euphratica* and prepared, then scanned and imaged by a Phenom Pro (Phenom, Netherlands) scanning electron microscope. Measurement was conducted in nine replicates.

Photosynthetic rates of heteromorphic leaves in different light intensities were measured by a LI-COR 6400 Portable Photosynthesis System (Li-Cor Inc., Lincoln, NE, USA) with an LED 2×3 red and blue light chamber. The light intensities of the measurements were separately set to 0, 50, 100, 200, 300, 400, 500, 700, 900, 1100, 1300, 1500, and 1800 μmoL·m^−2^·s^−1^. The solar radiation intensity on the leaf and the leaf temperature in the daytime were determined using a LI-COR 6400 Portable Photosynthesis System (Li-Cor Inc., Lincoln, NE, USA). Phytohormone extraction and purification of heteromorphic leaves were conducted according to the method of Atici et al. [78]. Then, extracts were injected into an Agilent 1100 HPLC-MS/MS system (Agilent Technologies, Böblingen, Germany) to determine auxin (IAA), abscisic acid (ABA), gibberellic acid (GA), and zeatin contents. Experiments were performed in five to nine replicates.

### 4.3. cDNA Library Construction and SEQUENCING

Total RNA was extracted from the two heteromorphic leaves of *P. euphratica* using Trizol reagent (Invitrogen, CA, USA) following themanufacturer’s instructions. A Bioanalyzer 2100 and an RNA 6000 Nano LabChip Kit (Agilent, Palo Alto, CA, USA) were used for testing the total RNA quantity and purity. Approximately 10μgof total RNA was used to deplete rRNA according to the instructionsfor the Epicentre Ribo-Zero Gold Kit (Illumina, San Diego, CA, USA). After purification, the poly(A)- or poly(A)+ RNA fractions were fragmented into small pieces using divalent cations. Then, the cleaved RNA fragments were reverse-transcribed to generate the final cDNA library following the protocol for the mRNA-Seq sample preparation kit (Illumina, San Diego, CA, USA). The average insert size for the paired-end libraries was 300 bp (±50 bp). After that, paired-end sequencing was conducted with anIllumina Hiseq 4000. Three biological repeats were used in the construction of the libraries.

### 4.4. Transcripts Assembly

First, Cutadapt [79], was used to remove the reads that contained adaptor contamination, undetermined bases, and low-quality bases. Sequence quality was then identified by FastQC (http://www.bioinformatics.babraham.ac.uk/projects/fastqc/). Bowtie2 [80] and topaht2 [81] were used to map reads to the genome of *P. euphratica* (https://www.ncbi.nlm.nih.gov/genome/?term=Populus%20euphratica). The assembly of mapped reads and the mergence of all transcriptomes from samples were both performed by using StringTie software [82]. After the transcriptome was generated, StringTie [82] and Ballgown [83] were used to evaluate the expression levels of all transcripts. All raw data of high-throughput sequencing have been deposited to the National Genomics Data Center (https://bigd.big.ac.cn) with the dataset accession number CRA002027.

### 4.5. lncRNA Identification

Firstly, transcripts less than 200 bp and transcripts that overlapped with known mRNAs were discarded. Then, the Coding Potential Calculator (CPC) package [84] and the Coding-Non-Coding Index (CNCI) tool [85] were applied to predict the coding potential of transcripts, so that transcripts with a CPC score <−1 and a CNCI score <0 were removed. Furthermore, the transcripts were filtered by the Coding-Potential Assessment Tool(CPAT) [86], with the default setting, and the Pfam [87] database, with an E-value <0.001. Finally, the remaining transcripts were considered as lncRNAs and categorized into different groups based on their genomic location.

### 4.6. Different Expression Analysis of mRNAs and lncRNAs

The expression level for mRNAs and lncRNAs was represented as FPKM (Fragments per kilobase of exon model per million mapped reads) [88] using StringTie [82]. The DEGs and DELs were confirmed with |log2 (fold change)| ≥1 and with statistical significance (*p* < 0.05) using the R package Ballgown [83].

### 4.7. Target Gene Prediction of lncRNAs and Establishment of Coexpression Networks

To explore the function of lncRNAs, in our study, DELs and DEGs were predicted as a cis–action relationship pair if they were no more than 100 kb genomic distance and coexpressed because lncRNAs can play a cis role acting on neighboring target genes [89].Further, trans–action relationship pairs between DELs and DEGs were identified based on sequence complementarity [90]. To visualize the interaction between lncRNAs and target protein-coding genes, Cytoscape software was used to establish the networks of lncRNAs and target genes [91].

### 4.8. Functional Classification of DEGs and the Target DEGs of DELs

Functional annotation was performed by GO (http://geneontology.org) annotation, by which genes were classified into biological process, molecular function, and cellular component classifications [92]. Then, GO terms were analyzed with GOseq software to obtain enriched GO terms [93]. The KEGG resource (http://www.genome.jp/kegg/) was adopted for functional classification of genes [94], after which KEGG pathway enrichment analysis was performed with KOBAS [95].

### 4.9. qRT-PCR Validation of lncRNA and Gene Expression Level

To verify the results of high-throughput RNA-seq, quantitative real-time polymerase chain reaction (qRT-PCR) was conducted. Total RNA was extracted from lanceolate and dentate broad-ovate leaves, separately, using the Plant RNA Kit (Beijing XinBaiAo biotechnology company, Beijing, China). Then, the sequence was reverse- transcribed into cDNA by FastQuant RT Super Mix (TIANGEN, Beijing, China). Ten lncRNAs and ten target genes were randomly selected to be verified; then, Primer Premier 5.0 software was adopted to design the gene-specific primers (GSPs). The reaction of qRT-PCR was performed with MiniOpticon Two-Color Real-Time PCR Detection System (BIO-RAD, USA), using SuperReal PreMix Plus (TIANGEN, Beijing, China). All reactions were carried out in three replicates, following two-step cycling conditions: 95 °C for 10 min, then 45 cycles of 95 °C for 10 s and 60 °C for 30 s. The histone superfamily protein H3 (*HIS*) and ribosomal L27e protein family (*RP*) were selected as the internal control for normalizing the results [96], and the lanceolate leaves were considered as the reference sample, the value of which was set to 1. The relative expression levels of candidates were calculated by the comparative cycle threshold method [97].

### 4.10. Statistical Analysis

Statistical Product and Service Solutions (SPSS) 19 program was used to conduct all statistical analyses of data, and data are presented as mean ± SD. The significant differences between means (*p* < 0.05) were examined by *t*-tests. Diagrams were produced by either Excel 2007 or OriginPro 8.0.

## 5. Conclusions

In this study, we analyzed morphological features coupled with lncRNA expression profiles and regulation of target genes of the two typical heteromorphic leaves, which develop in different layers of the canopy in *P. euphratica*. Our findings showed that, compared with lanceolate leaves, dentate broad-ovate leaves exhibit morphological traits in response to higher solar radiation. Furthermore, the interactions of candidate DELs and target DEGs associated with the response to environmental heterogeneity were determined, suggesting the greater tolerance to high-light or other adverse environmental conditions of dentate broad-ovate leaves and the advantage of lanceolate leaves in light capture for photosynthesis. In addition, *AUX/IAA* and *COL* genes might play important roles in the growth and development of heteromorphic leaves which may be affected by the light microenvironment in *P. euphratica*. These findings may provide new insights into heterophylly in plants, especially as they decipher the potential molecular mechanisms of environmental responses in heterophyllous woody plants.

## Figures and Tables

**Table 1:** Statistical data of the RNA-seq reads for the six libraries constructed from heteromorphic leaves of *P. euphratica* Oliv.

Sample	Raw Data		Valid Data		Q20%	Q30%	GC Content%
	Read	Base	Read	Base			
Lan_1	191446580	28.72G	135085944	20.26G	99.70	95.40	43.50
Lan_2	166688106	25.00G	112481178	16.87G	99.75	95.83	43.50
Lan_3	165831618	24.87G	110553910	16.58G	99.64	95.00	43.50
Db_1	163034286	24.46G	99461962	14.92G	99.68	95.73	44
Db_2	165850472	24.88G	111601846	16.74G	99.66	94.90	44
Db_3	172292388	25.84G	118538356	17.78G	99.65	95.60	44

Lan, lanceolate leaves; Db, dentate broad-ovate leaves; Q20%, proportion of the data for which quality values were greater than Q20 in the raw data; Q30%, proportion of the data for which quality values were greater than Q20 in the raw data.

**Table 2:** Statistics of genes and lncRNAs expressed in the six libraries constructed from heteromorphic leaves.

Sample	Lan_1	Lan_2	Lan_3	Db_1	Db_2	Db_3	Total
Expressed Gene	31,392	31,293	31,410	31,399	31,307	31,543	36,492
Expressed lncRNA	1095	1164	1166	1189	1153	1193	1725

Lan, lanceolate leaves; Db, dentate broad-ovate leaves.

## Supplementary Materials

Supplementary materials can be found at https://www.mdpi.com/1422-0067/20/20/5148/s1.
