# Supplementary material for: Conjoint Analysis of Genome-Wide lncRNA and mRNA Expression of Heteromorphic Leavesin Response to Environmental Heterogeneityin Populus euphratica
Source: Int J Mol Sci. 2019 Oct 17;20(20):5148. doi: 10.3390/ijms20205148 (PMC6829562; doi:10.3390/ijms20205148)
Supplement: Supplementary file 1 [file ijms-20-05148-s001.zip › supplementary materials.docx]

Figure A1


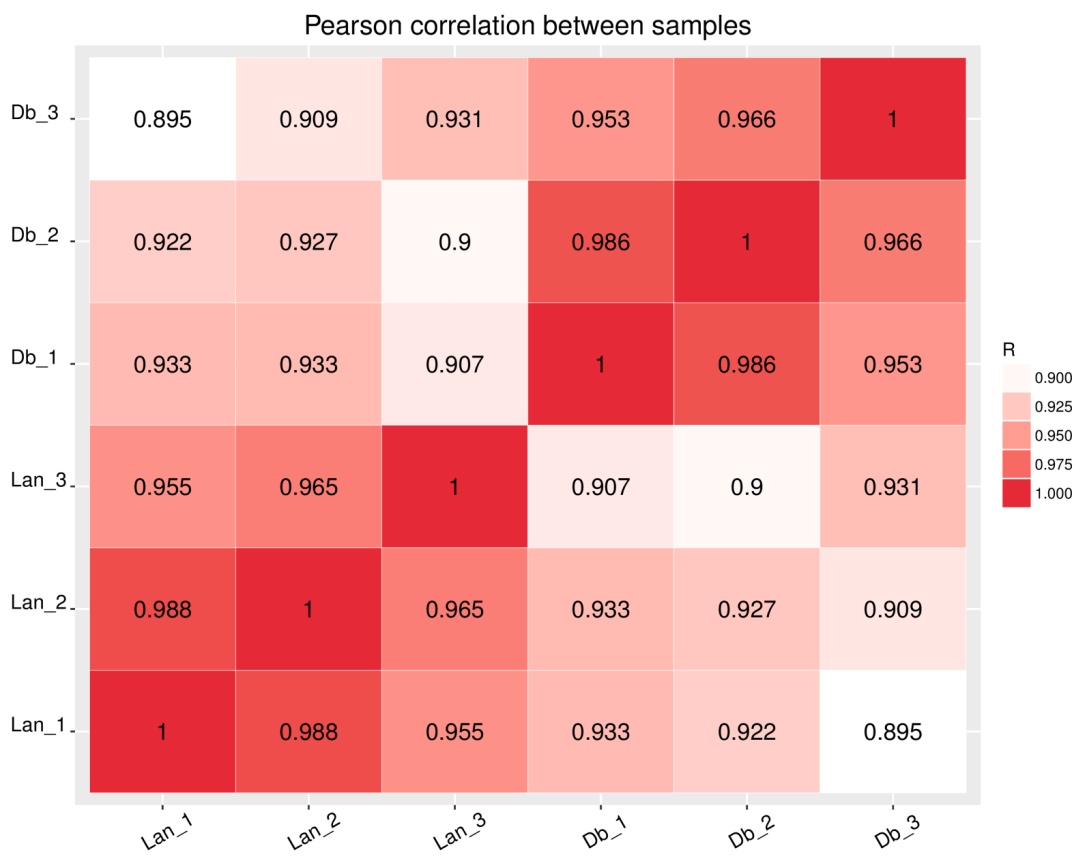


Figure A1. Pearson correlation between samples of RNA-Seq. Lan, lanceolate leaves; Db, dentate broad-ovate leaves.

Figure A2

**
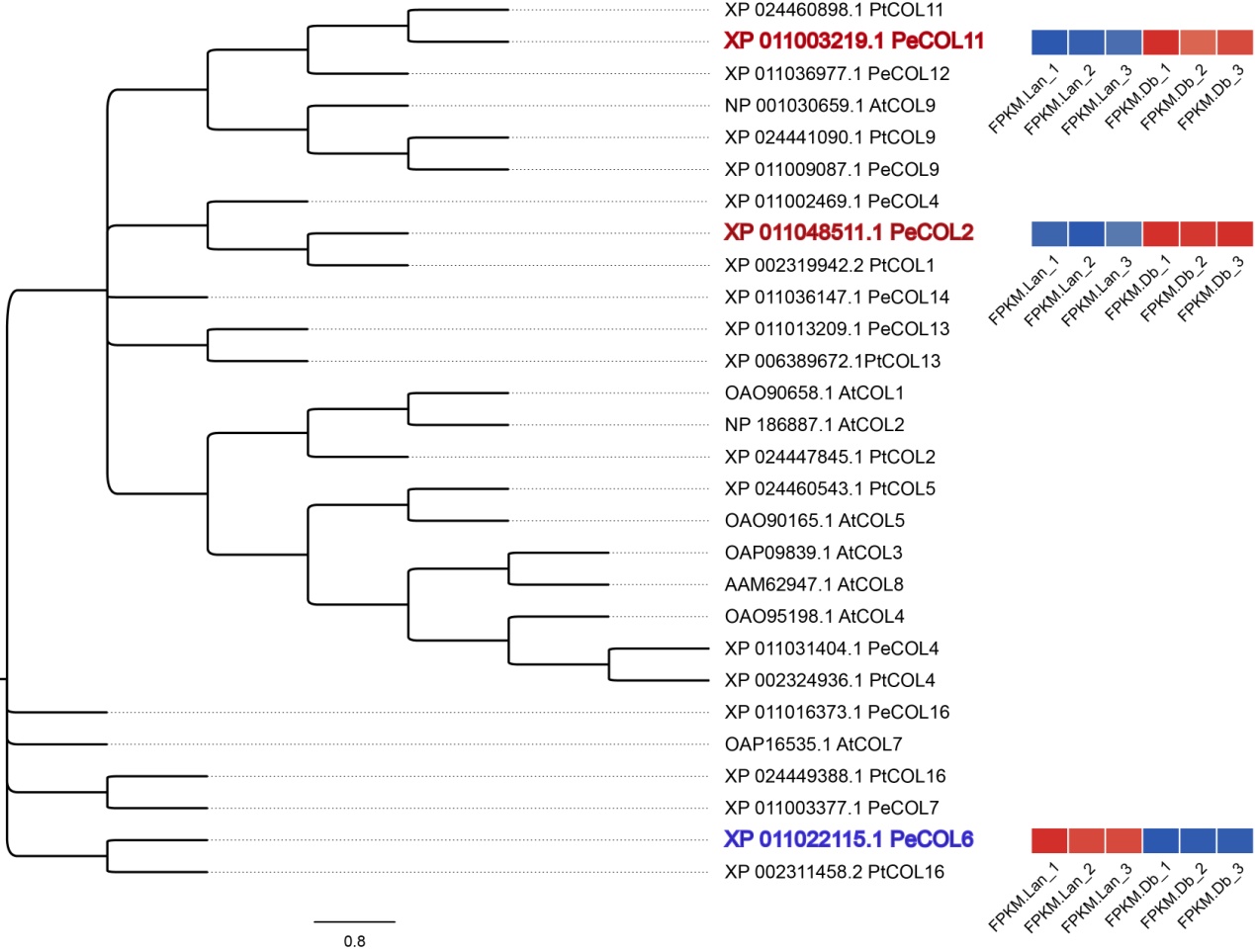
**

Figure A2. Phylogenetic tree of *PeCOL2*, *PeCOL11*, and *PeCOL6* genes. Heat map represented expression level of *PeCOL2*, *PeCOL11*, and *PeCOL6* genes in samples, phylogenetic tree was built by the MEGA 7.0 software via the neighbor-joining method, bootstrap analysis with 1000 replicates was performed to verify the stability of internal nodes.

Table S1

**Table S1.** The output of *t*-test for comparisons

1. *t*-test of comparison of lamina thickness in Figure 1 F:

| **Group Statistics** | | | | | | | | | | | | | | |  |  |  |
| --- | --- | --- | --- | --- | --- | --- | --- | --- | --- | --- | --- | --- | --- | --- | --- | --- | --- |
|  | VAR00001 | | N | Mean | | | | Std.Deviation | | | Std.Error Mean | | | |  |  |  |
| VAR00002 | Lan | | 9 | 242.8778 | | | | 4.76518 | | | 1.58839 | | | |  |  |  |
|  | Db | | 9 | 315.0222 | | | | 4.10420 | | | 1.36807 | | | |  |  |  |
| **Independent Samples Test** | | | | | | | | | | | | | | | | |  |
|  | | | | | Levene's Test for Equality of Variances | | | | | t-Test for Equality of Means | | | | | | |  |
|  |  |  |  |  | F | | Sig. | | | T | | | df | Sig. (2-tailed) | | |  |
|  |  |  |  |  |  |  |  |  |  |  |  |  |  |  |  |  |  |
| VAR00002 | Equal variance assumed | | | | .128 | | .725 | | | -34.415 | | | 16 | .000 | | |  |
|  | Equal variance not assumed | | | |  | |  | | | -34.415 | | | 15.656 | .000 | | |  |
| **Independent Samples Test** | | | | | | | | | | | | | | | | | |
|  | | | | | | t-Test for Equality of Means | | | | | | | | | | | |
|  |  |  |  |  |  | Mean Difference | | | Std.Error Difference | | | 95% Confidence Interval of the Difference | | | | | |
|  |  |  |  |  |  |  |  |  |  |  |  | Lower | | | | Upper | |
| VAR00002 | | Equal variance assumed | | | | -72.14444 | | | 2.09633 | | | -76.58847 | | | | -67.70042 | |
|  |  | Equal variance not assumed | | | | -72.14444 | | | 2.09633 | | | -76.59641 | | | | -67.69248 | |

2. *t*-test of comparison of specific leaf weight in Figure 1 G:

| **Group Statistics** | | | | | | | | | | | | | | |  |  |  |
| --- | --- | --- | --- | --- | --- | --- | --- | --- | --- | --- | --- | --- | --- | --- | --- | --- | --- |
|  | VAR00001 | | N | Mean | | | | Std.Deviation | | | Std.Error Mean | | | |  |  |  |
| VAR00002 | Lan | | 9 | 71.1934 | | | | 2.45522 | | | .81841 | | | |  |  |  |
|  | Db | | 9 | 82.3277 | | | | 3.17435 | | | 1.05812 | | | |  |  |  |
| **Independent Samples Test** | | | | | | | | | | | | | | | | |  |
|  | | | | | Levene's Test for Equality of Variances | | | | | t-Test for Equality of Means | | | | | | |  |
|  |  |  |  |  | F | | Sig. | | | T | | | df | Sig. (2-tailed) | | |  |
|  |  |  |  |  |  |  |  |  |  |  |  |  |  |  |  |  |  |
| VAR00002 | Equal variance assumed | | | | .946 | | .345 | | | -8.324 | | | 16 | .000 | | |  |
|  | Equal variance not assumed | | | |  | |  | | | -8.324 | | | 15.049 | .000 | | |  |
| **Independent Samples Test** | | | | | | | | | | | | | | | | | |
|  | | | | | | t-Test for Equality of Means | | | | | | | | | | | |
|  |  |  |  |  |  | Mean Difference | | | Std.Error Difference | | | 95% Confidence Interval of the Difference | | | | | |
|  |  |  |  |  |  |  |  |  |  |  |  | Lower | | | | Upper | |
| VAR00002 | | Equal variance assumed | | | | -11.13431 | | | 1.33769 | | | -13.97008 | | | | -8.29854 | |
|  |  | Equal variance not assumed | | | | -11.13431 | | | 1.33769 | | | -13.98472 | | | | -8.28391 | |

3. *t*-test of comparison of stomatal density in the adaxial epidermis in Figure 1 H:

| **Group Statistics** | | | | | | | | | | | | | | |  |  |  |
| --- | --- | --- | --- | --- | --- | --- | --- | --- | --- | --- | --- | --- | --- | --- | --- | --- | --- |
|  | VAR00001 | | N | Mean | | | | Std.Deviation | | | Std.Error Mean | | | |  |  |  |
| VAR00002 | Lan | | 9 | 93.8889 | | | | 4.64878 | | | 1.54959 | | | |  |  |  |
|  | Db | | 9 | 112.0000 | | | | 3.93700 | | | 1.31233 | | | |  |  |  |
| **Independent Samples Test** | | | | | | | | | | | | | | | | |  |
|  | | | | | Levene's Test for Equality of Variances | | | | | t-Test for Equality of Means | | | | | | |  |
|  |  |  |  |  | F | | Sig. | | | T | | | df | Sig. (2-tailed) | | |  |
|  |  |  |  |  |  |  |  |  |  |  |  |  |  |  |  |  |  |
| VAR00002 | Equal variance assumed | | | | 1.199 | | .290 | | | -8.919 | | | 16 | .000 | | |  |
|  | Equal variance not assumed | | | |  | |  | | | -8.919 | | | 15.578 | .000 | | |  |
| **Independent Samples Test** | | | | | | | | | | | | | | | | | |
|  | | | | | | t-Test for Equality of Means | | | | | | | | | | | |
|  |  |  |  |  |  | Mean Difference | | | Std.Error Difference | | | 95% Confidence Interval of the Difference | | | | | |
|  |  |  |  |  |  |  |  |  |  |  |  | Lower | | | | Upper | |
| VAR00002 | | Equal variance assumed | | | | -18.11111 | | | 2.03063 | | | -22.41585 | | | | -13.80637 | |
|  |  | Equal variance not assumed | | | | -18.11111 | | | 2.03063 | | | -22.41585 | | | | -13.79686 | |

4. *t*-test of comparison of stomatal density in the abaxial epidermis in Figure 1 H:

| **Group Statistics** | | | | | | | | | | | | | | |  |  |  |
| --- | --- | --- | --- | --- | --- | --- | --- | --- | --- | --- | --- | --- | --- | --- | --- | --- | --- |
|  | VAR00001 | | N | Mean | | | | Std.Deviation | | | Std.Error Mean | | | |  |  |  |
| VAR00002 | Lan | | 9 | 90.3333 | | | | 3.39116 | | | 1.13039 | | | |  |  |  |
|  | Db | | 9 | 103.5556 | | | | 4.77261 | | | 1.59087 | | | |  |  |  |
| **Independent Samples Test** | | | | | | | | | | | | | | | | |  |
|  | | | | | Levene's Test for Equality of Variances | | | | | t-Test for Equality of Means | | | | | | |  |
|  |  |  |  |  | F | | Sig. | | | T | | | df | Sig. (2-tailed) | | |  |
|  |  |  |  |  |  |  |  |  |  |  |  |  |  |  |  |  |  |
| VAR00002 | Equal variance assumed | | | | 2.088 | | .168 | | | -6.775 | | | 16 | .000 | | |  |
|  | Equal variance not assumed | | | |  | |  | | | -6.775 | | | 14.437 | .000 | | |  |
| **Independent Samples Test** | | | | | | | | | | | | | | | | | |
|  | | | | | | t-Test for Equality of Means | | | | | | | | | | | |
|  |  |  |  |  |  | Mean Difference | | | Std.Error Difference | | | 95% Confidence Interval of the Difference | | | | | |
|  |  |  |  |  |  |  |  |  |  |  |  | Lower | | | | Upper | |
| VAR00002 | | Equal variance assumed | | | | -13.22222 | | | 1.95157 | | | -17.35937 | | | | -9.08507 | |
|  |  | Equal variance not assumed | | | | -13.22222 | | | 1.95157 | | | -17.39607 | | | | -9.04837 | |

5. *t*-test of comparison of stomatal length in the adaxial epidermis in Figure 1 I:

| **Group Statistics** | | | | | | | | | | | | | | |  |  |  |
| --- | --- | --- | --- | --- | --- | --- | --- | --- | --- | --- | --- | --- | --- | --- | --- | --- | --- |
|  | VAR00001 | | N | Mean | | | | Std.Deviation | | | Std.Error Mean | | | |  |  |  |
| VAR00002 | Lan | | 9 | 20.3222 | | | | .51667 | | | .17222 | | | |  |  |  |
|  | Db | | 9 | 19.3667 | | | | .39051 | | | .13017 | | | |  |  |  |
| **Independent Samples Test** | | | | | | | | | | | | | | | | |  |
|  | | | | | Levene's Test for Equality of Variances | | | | | t-Test for Equality of Means | | | | | | |  |
|  |  |  |  |  | F | | Sig. | | | T | | | df | Sig. (2-tailed) | | |  |
|  |  |  |  |  |  |  |  |  |  |  |  |  |  |  |  |  |  |
| VAR00002 | Equal variance assumed | | | | 1.512 | | .237 | | | 4.426 | | | 16 | .000 | | |  |
|  | Equal variance not assumed | | | |  | |  | | | 4.426 | | | 14.891 | .000 | | |  |
| **Independent Samples Test** | | | | | | | | | | | | | | | | | |
|  | | | | | | t-Test for Equality of Means | | | | | | | | | | | |
|  |  |  |  |  |  | Mean Difference | | | Std.Error Difference | | | 95% Confidence Interval of the Difference | | | | | |
|  |  |  |  |  |  |  |  |  |  |  |  | Lower | | | | Upper | |
| VAR00002 | | Equal variance assumed | | | | .95556 | | | .21588 | | | .49791 | | | | 1.41320 | |
|  |  | Equal variance not assumed | | | | .95556 | | | .21588 | | | .49512 | | | | 1.41599 | |

6. *t*-test of comparison of stomatal length in the abaxial epidermis in Figure 1 I:

| **Group Statistics** | | | | | | | | | | | | | | |  |  |  |
| --- | --- | --- | --- | --- | --- | --- | --- | --- | --- | --- | --- | --- | --- | --- | --- | --- | --- |
|  | VAR00001 | | N | Mean | | | | Std.Deviation | | | Std.Error Mean | | | |  |  |  |
| VAR00002 | Lan | | 9 | 20.3556 | | | | .62071 | | | .20690 | | | |  |  |  |
|  | Db | | 9 | 19.3333 | | | | .41833 | | | .13944 | | | |  |  |  |
| **Independent Samples Test** | | | | | | | | | | | | | | | | |  |
|  | | | | | Levene's Test for Equality of Variances | | | | | t-Test for Equality of Means | | | | | | |  |
|  |  |  |  |  | F | | Sig. | | | T | | | df | Sig. (2-tailed) | | |  |
|  |  |  |  |  |  |  |  |  |  |  |  |  |  |  |  |  |  |
| VAR00002 | Equal variance assumed | | | | 3.887 | | .066 | | | 4.097 | | | 16 | .000 | | |  |
|  | Equal variance not assumed | | | |  | |  | | | 4.097 | | | 14.025 | .000 | | |  |
| **Independent Samples Test** | | | | | | | | | | | | | | | | | |
|  | | | | | | t-Test for Equality of Means | | | | | | | | | | | |
|  |  |  |  |  |  | Mean Difference | | | Std.Error Difference | | | 95% Confidence Interval of the Difference | | | | | |
|  |  |  |  |  |  |  |  |  |  |  |  | Lower | | | | Upper | |
| VAR00002 | | Equal variance assumed | | | | 1.02222 | | | .24951 | | | .49329 | | | | 1.55115 | |
|  |  | Equal variance not assumed | | | | 1.02222 | | | .24951 | | | .48717 | | | | 1.55727 | |

7. *t*-test of comparison of IAA content in Figure 2 D:

| **Group Statistics** | | | | | | | | | | | | | | |  |  |  |
| --- | --- | --- | --- | --- | --- | --- | --- | --- | --- | --- | --- | --- | --- | --- | --- | --- | --- |
|  | VAR00001 | | N | Mean | | | | Std.Deviation | | | Std.Error Mean | | | |  |  |  |
| VAR00002 | Lan | | 6 | 6.5426 | | | | .48966 | | | .19990 | | | |  |  |  |
|  | Db | | 6 | 8.4483 | | | | .48679 | | | .19873 | | | |  |  |  |
| **Independent Samples Test** | | | | | | | | | | | | | | | | |  |
|  | | | | | Levene's Test for Equality of Variances | | | | | t-Test for Equality of Means | | | | | | |  |
|  |  |  |  |  | F | | Sig. | | | T | | | df | Sig. (2-tailed) | | |  |
|  |  |  |  |  |  |  |  |  |  |  |  |  |  |  |  |  |  |
| VAR00002 | Equal variance assumed | | | | .012 | | .916 | | | -6.761 | | | 10 | .000 | | |  |
|  | Equal variance not assumed | | | |  | |  | | | -6.761 | | | 10.000 | .000 | | |  |
| **Independent Samples Test** | | | | | | | | | | | | | | | | | |
|  | | | | | | t-Test for Equality of Means | | | | | | | | | | | |
|  |  |  |  |  |  | Mean Difference | | | Std.Error Difference | | | 95% Confidence Interval of the Difference | | | | | |
|  |  |  |  |  |  |  |  |  |  |  |  | Lower | | | | Upper | |
| VAR00002 | | Equal variance assumed | | | | -1.90570 | | | .28188 | | | -2.53376 | | | | -1.27764 | |
|  |  | Equal variance not assumed | | | | -1.90570 | | | .28188 | | | -2.53377 | | | | -1.27763 | |

8. *t*-test of comparison of ABA content in Figure 2 E:

| **Group Statistics** | | | | | | | | | | | | | | |  |  |  |
| --- | --- | --- | --- | --- | --- | --- | --- | --- | --- | --- | --- | --- | --- | --- | --- | --- | --- |
|  | VAR00001 | | N | Mean | | | | Std.Deviation | | | Std.Error Mean | | | |  |  |  |
| VAR00002 | Lan | | 6 | 167.1389 | | | | 3.00248 | | | 1.22576 | | | |  |  |  |
|  | Db | | 6 | 166.4361 | | | | 5.97055 | | | 2.43746 | | | |  |  |  |
| **Independent Samples Test** | | | | | | | | | | | | | | | | |  |
|  | | | | | Levene's Test for Equality of Variances | | | | | t-Test for Equality of Means | | | | | | |  |
|  |  |  |  |  | F | | Sig. | | | T | | | df | Sig. (2-tailed) | | |  |
|  |  |  |  |  |  |  |  |  |  |  |  |  |  |  |  |  |  |
| VAR00002 | Equal variance assumed | | | | 6.365 | | .030 | | | .258 | | | 10 | .802 | | |  |
|  | Equal variance not assumed | | | |  | |  | | | .258 | | | 7.377 | .804 | | |  |
| **Independent Samples Test** | | | | | | | | | | | | | | | | | |
|  | | | | | | t-Test for Equality of Means | | | | | | | | | | | |
|  |  |  |  |  |  | Mean Difference | | | Std.Error Difference | | | 95% Confidence Interval of the Difference | | | | | |
|  |  |  |  |  |  |  |  |  |  |  |  | Lower | | | | Upper | |
| VAR00002 | | Equal variance assumed | | | | .70282 | | | 2.72832 | | | -5.37625 | | | | 6.78189 | |
|  |  | Equal variance not assumed | | | | .70282 | | | 2.72832 | | | -5.68241 | | | | 7.08804 | |

9. *t*-test of comparison of GA content in Figure 2 F:

| **Group Statistics** | | | | | | | | | | | | | | |  |  |  |
| --- | --- | --- | --- | --- | --- | --- | --- | --- | --- | --- | --- | --- | --- | --- | --- | --- | --- |
|  | VAR00001 | | N | Mean | | | | Std.Deviation | | | Std.Error Mean | | | |  |  |  |
| VAR00002 | Lan | | 6 | 297.9372 | | | | 6.71029 | | | 2.73946 | | | |  |  |  |
|  | Db | | 6 | 299.5897 | | | | 22.40756 | | | 9.14785 | | | |  |  |  |
| **Independent Samples Test** | | | | | | | | | | | | | | | | |  |
|  | | | | | Levene's Test for Equality of Variances | | | | | t-Test for Equality of Means | | | | | | |  |
|  |  |  |  |  | F | | Sig. | | | T | | | df | Sig. (2-tailed) | | |  |
|  |  |  |  |  |  |  |  |  |  |  |  |  |  |  |  |  |  |
| VAR00002 | Equal variance assumed | | | | 11.953 | | .006 | | | -.173 | | | 10 | .866 | | |  |
|  | Equal variance not assumed | | | |  | |  | | | -.173 | | | 5.890 | .868 | | |  |
| **Independent Samples Test** | | | | | | | | | | | | | | | | | |
|  | | | | | | t-Test for Equality of Means | | | | | | | | | | | |
|  |  |  |  |  |  | Mean Difference | | | Std.Error Difference | | | 95% Confidence Interval of the Difference | | | | | |
|  |  |  |  |  |  |  |  |  |  |  |  | Lower | | | | Upper | |
| VAR00002 | | Equal variance assumed | | | | -1.65252 | | | 9.54923 | | | -22.92953 | | | | 19.62449 | |
|  |  | Equal variance not assumed | | | | -1.65252 | | | 9.54923 | | | -25.12519 | | | | 21.82015 | |

10. *t*-test of comparison of Zeatin content in Figure 2 G:

| **Group Statistics** | | | | | | | | | | | | | | |  |  |  |
| --- | --- | --- | --- | --- | --- | --- | --- | --- | --- | --- | --- | --- | --- | --- | --- | --- | --- |
|  | VAR00001 | | N | Mean | | | | Std.Deviation | | | Std.Error Mean | | | |  |  |  |
| VAR00002 | Lan | | 6 | .5979 | | | | .04205 | | | .01717 | | | |  |  |  |
|  | Db | | 6 | .5769 | | | | .02159 | | | .00882 | | | |  |  |  |
| **Independent Samples Test** | | | | | | | | | | | | | | | | |  |
|  | | | | | Levene's Test for Equality of Variances | | | | | t-Test for Equality of Means | | | | | | |  |
|  |  |  |  |  | F | | Sig. | | | T | | | df | Sig. (2-tailed) | | |  |
|  |  |  |  |  |  |  |  |  |  |  |  |  |  |  |  |  |  |
| VAR00002 | Equal variance assumed | | | | 3.546 | | .089 | | | 1.084 | | | 10 | .304 | | |  |
|  | Equal variance not assumed | | | |  | |  | | | 1.084 | | | 7.465 | .312 | | |  |
| **Independent Samples Test** | | | | | | | | | | | | | | | | | |
|  | | | | | | t-Test for Equality of Means | | | | | | | | | | | |
|  |  |  |  |  |  | Mean Difference | | | Std.Error Difference | | | 95% Confidence Interval of the Difference | | | | | |
|  |  |  |  |  |  |  |  |  |  |  |  | Lower | | | | Upper | |
| VAR00002 | | Equal variance assumed | | | | .02092 | | | .01930 | | | -.02208 | | | | .06392 | |
|  |  | Equal variance not assumed | | | | .02092 | | | .01930 | | | -.02415 | | | | .06598 | |

Table S2

**Table S2.** Mapping data of RNA-seq reads for the six libraries constructed from heteromorphic leaves of *Populus euphratica* Oliv.

| Sample | Valid reads | Mapped reads | Unique Mapped reads | Reads map to sense strand | Reads map to antisense strand |
| --- | --- | --- | --- | --- | --- |
| Lan_1 | 82720534 | 62939777  (76.09%) | 56341432  (68.11%) | 28282684  (34.19%) | 28058748  (33.92%) |
| Lan_2 | 70184426 | 52761346  (75.18%) | 47261935  (67.34%) | 23720994  (33.80%) | 23540941  (33.54%) |
| Lan_3 | 72166248 | 54593354  (75.65%) | 48936345  (67.81%) | 24568493  (34.04%) | 24367852  (33.77%) |
| Db_1 | 58231830 | 44711708  (76.78%) | 40384534  (69.35%) | 20268348  (34.81%) | 20116186  (34.55%) |
| Db_2 | 60109234 | 45007660  (74.88%) | 40534878  (67.44%) | 20333389  (33.83%) | 20201489  (33.61%) |
| Db_3 | 66548602 | 49775063  (74.80%) | 44577462  (66.98%) | 22368539  (33.61%) | 22208923  (33.37%) |

Lan, lanceolate leaves; Db, dentate broad-ovate leaves.

Table S3

**Table S3.** Specific primer pairs used for RT-qPCR expression analysis

| Candidate genes |  | Primer sequences (5'-3') |
| --- | --- | --- |
| LOC105121245 | Forward | GGGCATAGCAATGTGGAGG |
|  | Reverse | TGAAAGCCGAATCTAGGAACG |
| LOC105112019 | Forward | CCTTGCTTGGGGATTCTGT |
|  | Reverse | ACTGGTCACTGGCTTTGTCTG |
| LOC105113472 | Forward | CTCCTTCGGTTCGCTCCT |
|  | Reverse | ACCACGGTGTTGCCCATT |
| LOC105116063 | Forward | ACCACCAAGCCTGTTTCC |
|  | Reverse | TGTCCCAGCCATAATCACC |
| LOC105122307 | Forward | TGGGGAAACCACCCTAAAA |
|  | Reverse | ACCGCCGTCAGCAACAAAC |
| LOC105116349 | Forward | GCCAGGAGTAAAGGTGAA |
|  | Reverse | ATGACAGGGACAACGAAT |
| LOC105129495 | Forward | ACTTCTGTGCTAGGTGCCG |
|  | Reverse | GCCAGTGCTTGCCTTCTC |
| LOC105142529 | Forward | CAGATGATATGGAGGAAGAT |
|  | Reverse | GAATACGAACGGTTGAGA |
| LOC105110021 | Forward | ATTTGGTCGTCGGTTCTT |
|  | Reverse | CCCTCATTATCCCTTTGTT |
| LOC105123992 | Forward | GACCCTGATGAATGTTGG |
|  | Reverse | CTGGATACTCGTGCCTCT |
| Candidate LncRNAs |  |  |
| MSTRG.20637.1 | Forward | GAATGCGAGGTTTTGTGAGTG |
|  | Reverse | CAGAACAGAAGCCCTGGAAG |
| MSTRG.25621.1 | Forward | GAGTGGAAACCTTGTGGTCGTA |
|  | Reverse | AGGGCTTTCTGCGGATGA |
| MSTRG.5613.1 | Forward | TTTCGTCTTCCAAACAATG |
|  | Reverse | CTCCTTCCTTTCCCTCTG |
| MSTRG.21947.1 | Forward | CGAGGGAAGGATTGTTGG |
|  | Reverse | TGGATTTGCTCTTGGGTT |
| MSTRG.17663.1 | Forward | ACCACAAACAGGCACCCAA |
|  | Reverse | TTTCCTCCGTCACTGTAAACCT |
| MSTRG.20656.2 | Forward | GAGTGATGGAAGGAGATAGGGG |
|  | Reverse | TCGTATTGGAGATGTGAGTGAGA |
| MSTRG.21859.1 | Forward | TCAAGTGTTGGTCCTGTT |
|  | Reverse | CAAATCCCTGGTGTTCTA |
| MSTRG.20656.5 | Forward | GAAGGTGGAGCGATAAGC |
|  | Reverse | CCTGTCATGGCAAAGGTC |
| MSTRG.4696.1 | Forward | TACTTTATGAGAACCCTGTGC |
|  | Reverse | CCACCTCCCTTCACTTCA |
| MSTRG.3335.1  MSTRG.4123.1  MSTRG.4123.1 | Forward | ATCCCAATACATTTCTCCA |
| MSTRG.4123.1 | Reverse | CCGTTACATCATCATCCC |
